# Diseases of Animals in 1904

**Published:** 1905-01-21

**Authors:** 


### Diseases of Animals in 1904.

The diseases of the lower animals have many features of interest to those engaged in medical and surgical practice. Comparative pathology has already suggested solutions for some of the problems presented by disease processes, as these are found in human beings, and barely more than the fringe of the subject has yet been touched. It is probable that to an increasing extent mankind will in the future gain aid in the study of its own pathological misfortunes by investigating the similar incidents which afflict our "poor relations." But beyond the relationship thus suggested, the diseases of the lower animals, and more particularly those which are apt to occur in epidemics and to attack animals having close associations with man's domestic or business concerns, have important social and economic interests. This is in part due to the fact that some of these diseases may be communicated from the lower animals to man himself, and it is indeed not improbable that this occurs over a much wider area than is generally suspected. Again, it has to be remembered that in the case of animals used for human food there is not only the possibility that these when slaughtered may be the means of spreading disease, but this fact brings with it the proposal for municipal control and adequate inspection of meat supplies. And yet once more, from these there arise questions of permitting or prohibiting the importation of food stuffs and living or slaughtered animals from particular countries, the prohibition, if decided on, necessarily increasing prices by diminishing supplies. Hence the study of the diseases, and more particularly the study of the epidemic diseases, so far from being a mere opportunity for the pathologist, has on its very threshold such important questions as the food supply, the health and the economic interests of the nation. From these points of view it is satisfactory to know that the incidence and development of the diseases in question are kept under careful official observation, and a summary of the weekly returns for 1904 which recently appeared in the *Times* shows some of the interesting and valuable results which accrue from this system. First may be noted the entire absence of rabies from Great Britain, no case having been reported since 1902, in which year thirteen were recorded. Even more satisfactory is it to know that pleuro-pneumonia has been absent from this country since 1898. This is a striking contrast to the state of affairs existing during 1870-80 when the annual outbreaks of this disease, causing much financial loss and business inconvenience, were never less than a thousand and in one year exceeded three thousand. Foot-and-mouth disease is another instance in which 1904 can boast a clean bill of health. Of swine fever the account is much less cheering, the number of outbreaks during the year reaching a total of 1,196 and involving the slaughter of 5,603 animals. Still, these figures are the lowest which have been recorded in connection with this disease during the last eleven years, and possibly some share in this result is to be attributed to the fact that at the end of 1893 the Diseases of Animals Act passed from the administration of the local authorities into the hands of the Board of Agriculture. As opposed to these favourable figures such serious diseases as anthrax and glanders show a decided increase. Of anthrax during 1904 there were 1,053 outbreaks reported, and the number of animals attacked was 1,570. These totals exceed the record of any single year since the issue of the Anthrax Order in 1886, and certainly suggest widespread ignorance of the effective method of dealing with this disease. The figures for glanders show a total of 1,535 recorded outbreaks, the number of horses attacked being 2,658. For the great majority of these outbreaks the metropolitan area is responsible, no less than 1,080 having occurred in the county of London, and 192 in the neighbouring counties of Middlesex, Essex, and Surrey. Reviewing these figures it is evident that, while much has been accomplished, there is yet room for the further development of pathological and bacteriological science in the prevention of diseases which are prone to attack horses and cattle and thus to prejudice the economic interests of the community.

